# Exploring stakeholders’ experiences of comprehensive geriatric assessment in the community and out-patient settings: a qualitative evidence synthesis

**DOI:** 10.1186/s12875-023-02222-2

**Published:** 2023-12-13

**Authors:** Christina Hayes, Christine Fitzgerald, Íde O’Shaughnessy, Brian Condon, Aoife Leahy, Margaret O’Connor, Molly Manning, Anne Griffin, Liam Glynn, Katie Robinson, Rose Galvin

**Affiliations:** 1 School of Allied Health, Faculty of Education and Health Sciences, Ageing Research Centre, Health Research Institute, University of Limerick, Limerick, Ireland; 2 Department of Ageing and Therapeutics, University Hospital Limerick, Dooradoyle, Limerick, Ireland; 3 School of Medicine, Faculty of Education and Health Sciences, University of Limerick, Limerick, Ireland; 4 HRB Primary Care Clinical Trials Network Ireland, Discipline of General Practice, School of Medicine, HRB Primary Care Clinical Trials Network Ireland, National University of Ireland Galway, Galway, Ireland

**Keywords:** Older adults, Comprehensive geriatric assessment, Community setting, Primary care, Out-patient, Qualitative evidence synthesis, Meta-ethnography

## Abstract

**Background:**

Comprehensive Geriatric Assessment (CGA) is a multidimensional interdisciplinary process that addresses an older adult’s biopsychosocial capabilities to create an integrated and co-ordinated plan of care. While quantitative evidence that demonstrates the positive impacts of CGA on clinical and process outcomes has been synthesised, to date qualitative research reporting how older adults and service providers experience CGA has not been synthesised. This study aimed to systematically review and synthesise qualitative studies reporting community-dwelling older adults’, caregivers’ and healthcare professionals’ (HCP) experiences of CGA in the primary care and out-patient (OPD) setting.

**Method:**

We systematically searched five electronic databases including MEDLINE, CINAHL, PsycINFO, PsycARTICLES and Social Sciences Full Text targeting qualitative or mixed methods studies that reported qualitative findings on older adults’, caregivers’ and HCPs’ experiences of CGA in primary care or out-patient settings. There were no language or date restrictions applied to the search. The protocol was registered with the PROSPERO database (Registration: CRD42021283167). The methodological quality of the included studies was appraised using the Critical Appraisal Skills Programme checklist for qualitative research. Results were synthesised according to Noblit and Hare’s seven-step approach to meta-ethnography, which involves an iterative and inductive process of data synthesis.

**Results:**

Fourteen studies were included where CGA was completed in the home, general practice, out-patient setting in acute hospitals and in hybrid models across the community and hospital-based OPD settings. Synthesis generated four key themes: (1) CGA is experienced as a holistic process, (2) The home environment enhances CGA, (3) CGA in the community is enabled by a collaborative approach to care, and (4) Divergent experiences of the meaningful involvement of older adults, caregivers and family in the CGA process.

**Conclusion:**

Findings demonstrate that CGA in a home-based or OPD setting allows for a holistic and integrated approach to care for community-dwelling older adults while increasing patient satisfaction and accessibility of healthcare. Healthcare professionals in the community should ensure meaningful involvement of older adults and their families or caregivers in the CGA process. Further robustly designed and well reported trials of different models of community-based CGA informed by the findings of this synthesis are warranted.

**Supplementary Information:**

The online version contains supplementary material available at 10.1186/s12875-023-02222-2.

## Introduction

There is growing global recognition of a need to shift healthcare delivery for older adults from reactive, episodic hospital-based management of care towards an integrated, proactive and preventative approach in the community setting [1]. The World Health Organization (WHO) advocates for a shift towards a comprehensive community-based approach to care addressing the complex profile of this population that aims to prevent declines in intrinsic capacity and foster healthy ageing [2, 3].

Comprehensive Geriatric Assessment (CGA) is considered a pillar of geriatric care [4–6] and a key component in the delivery of integrated clinical care to older adults [7]. CGA is a “multidimensional interdisciplinary diagnostic process focused on determining a frail elderly person’s medical, psychological and functional capability in order to develop a coordinated and integrated plan for treatment and long term follow up” [8]. While substantial evidence supports the effectiveness of CGA for older adults, a lack of clarity exists surrounding the setting in which CGA is conducted with evidence indicating conflicting results across settings [5, 9, 10]. A Cochrane review of 29 trials synthesised evidence on the effectiveness and resource use of CGA for older adults admitted to hospital [5]. Findings demonstrated an increase in the likelihood of older adults being alive and living in their own homes after an emergency admission to hospital (risk ratio (RR) 1.06, 95% confidence interval (CI) 1.01 to 1.10) and reduced the likelihood of nursing home (NH) admission (RR 0.80, 95% CI 0.72 to 0.89) at three to 12 months’ follow-up [5]. Limited high quality evidence exists to support the effectiveness of CGA in the emergency department (ED) for older adults living with frailty [10]. However, CGA carried out in an acute geriatric unit demonstrated positive effects on clinical and process outcomes for older adults with acute medical complaints [11]. In contrast, a recent Cochrane review of 21 studies examining CGA conducted in the participant's home, general practice or community-based clinic demonstrated little or no impact on mortality (RR 0.88, 95% CI 0.76 to 1.02), or NH admission (RR 0.93, 95% CI 0.76 to 1.14), but did report low-certainty evidence that it reduces the risk of unplanned hospital admission at 14-month follow-up (RR 0.83, 95% CI 0.70 to 0.99)[9]. Authors concluded however that further exploration of the satisfaction and experience of older adults who undergo CGA in the community setting is required.

Research around CGA has largely focused on quantitative syntheses of the international evidence across acute and primary care settings [5, 9]. However, there is no qualitative evidence synthesis that explores service user and service provider experiences of CGA in the community and Out-Patient Department (OPD) setting to date. Qualitative health research offers an opportunity to compliment quantitative research by capturing the rounded complexity of the lived experiences of people across social, cultural and political contexts [12]. It facilitates an understanding of pathways of care [13] and is instrumental to identifying key factors to implementation of healthcare through engagement of service users and service providers [14], which is valuable in exploring the recent shift of healthcare delivery towards the community-setting. To date, few studies have synthesised the totality of evidence in relation to service user and service provider experience of CGA. A forthcoming Qualitative Evidence Synthesis (QES) by O’Shaughnessy and colleagues explored stakeholder perspectives in the acute in-patient setting and found that the acute setting is not an ideal environment for patient involvement or care planning for older adults (O’Shaughnessy, personal communication). Additionally, a systematic integrative review by Sum and colleagues’ synthesised the literature on quantitative health outcomes and qualitative implementation barriers and facilitators of CGA [15]. It was reported that a lack of communication and a reluctance to providing preventative care were barriers to implementation while the use of skilled staff, patient education and care coordination were seen as facilitators to CGA [15]. However, this review only included studies that addressed implementation barriers and facilitators to CGA.

This study aims to systematically search and synthesise the available qualitative literature exploring older adults’, healthcare professionals’ (HCP) and caregivers’ experiences of CGA in community and OPD settings. This QES will be informative for policy and future development of community-based services for the growing population of older adults globally.

## Methods

### Study design

This qualitative evidence synthesis followed Noblit and Hare’s seven step approach to a meta-ethnography [16]. This study was registered on the PROSPERO database (Registration: CRD42021283167) and is reported in accordance with the eMERGE Reporting Guidance [17](Additional file 1).

Noblit and Hare’s approach involves an iterative, interpretive and inductive process by translating studies into one another, leading to the creation of novel interpretations of the phenomenon being explored from primary qualitative studies [17, 18]. Through comparison of concepts of individual studies, a conceptual richness can be achieved allowing the generation of new understanding [19, 20]. Schütz’s concept of first-, second-, and third-order constructs was used, whereby first order constructs represent original quotations from study participants, second-order constructs represent researcher interpretations of original data, from which, through meta-level synthesis authors derive third-order constructs [21]. Third-order constructs offer more than a traditional literature review, by underpinning the findings from the included studies but also extending beyond them.

#### Step 1

The first step involved identification of the research gap and refinement of the research question; “What are older adults’, caregivers’ and HCPs’ experiences and perspectives of CGA in community and OPD settings?”.

#### Step 2

This step focused on selecting studies for inclusion in the synthesis and involved a systematic search, screening and quality appraisal of potential studies. A systematic search of five electronic databases (MEDLINE, CINAHL, PsycINFO, PsycARTICLES and Social Sciences Full Text) using “comprehensive geriatric assessment” and “qualitative research” as keywords in conjunction with MeSH terms was conducted in May 2023. The search strategy is available in Additional file 2. There were no date restrictions applied. The search was limited to peer-reviewed publications i.e., grey literature and abstracts were excluded, and studies were limited to those published in English. Included studies from a recent systematic review were hand searched [15]. Reference lists of included studies were also searched for additional papers.

Studies that reported qualitative methods of data collection and analysis and focused on community-dwelling older adults’, caregivers’ and healthcare professionals’ experiences of CGA in community or OPD settings were included. Parker and colleagues’ definition of CGA as a ‘multidimensional, multidisciplinary process, which identifies medical, social and functional needs, and the development of an integrated/co-ordinated care plan to meet those needs’ was used to determine studies for inclusion in this review [22]. Studies reporting mixed methods were also included where qualitative data was reported regarding stakeholder experiences of CGA and where data could be extracted separately. All references were imported into Endnote X9 software [23] where duplicates were subsequently removed. Titles and abstracts were independently screened for eligibility by two authors (ÍO’S & KR). Any inconsistencies in the inclusion process were resolved via consensus or third author consultation (RG). Two reviewers (CH and CF) independently read the full-text articles identified for inclusion. A third reviewer (KR) was consulted when consensus could not be reached regarding final full-text inclusion in the review. This occurred in one instance and the study was included in the QES. The methodological quality of included papers were appraised independently by two authors (CH and CF) using Critical Appraisal Skills Program (CASP) Checklist [24]. Any discrepancies were resolved by consensus or discussion with a third author (KR).

#### Step 3

This step involved the iterative process of closely and carefully reading the included articles in a repetitive and active manner while also identifying the main concepts. The term ‘concept’ was defined by the researchers as ‘a meaningful idea that develops by comparing particular instances’ [25]. Important study characteristics to contextualise findings were extracted by CH using a custom Excel file template and the extracted data were checked by CF (Table 1). Articles were initially read by two authors (CH and CF) who noted key concepts. Each author maintained a reflective journal to reflexively consider their impact on all stages of the research process [26]. First and second order constructs were extracted into NVivo Version 12 Pro software [27] to assist with data management (CH and CF) and discussed amongst the team (CH, CF, KR and I’OS). To ensure sufficient depth and richness of extraction, data extraction and coding were completed on two studies which was reviewed by the team prior to completion on all studies.

**Table 1:** Descriptive characteristics of included studies

Citation and country	Title	Population of participants undergoing CGA	Stakeholders involved in qualitative component	Setting of the CGA	Aim(s)	Methods	Description of the CGA process	Team composition	Specialist training
Barkhausen et al. 2015. Germany [40]	“It’s MAGIC”—development of a manageable geriatric assessment for general practice use	Older adults aged ≥ 72 years	General practitioner (*n* = 20)	OPD in General Practice	To develop a “manageable geriatric assessment – MAGIC”, specially tailored to the requirements of daily primary care	Mixed methods. Qualitative focus groups. Mind-mapping analysis	A brief comprehensive screening tool to facilitate identification of unidentified health problems in primary care amongst multimorbid older people	Not reported	Not reported
Berkhout‐Byrne et al. 2023. The Netherlands [33]	Nephrology‐tailored geriatric assessment as decision‐making tool in kidney failure	Older people living at home aged ≥ 65 years, with chronic kidney disease stage G4‐G5, or a recent kidney transplantation	Older people (*n* = 18). Caregivers (*n* = 4). HCPs (*n* = 25)	Out-patient clinic in acute hospital setting	To explore the perspectives of patients and healthcare professionals on nephrology‐tailored geriatric assessment to fuel decision‐making for treatment choices in older patients with kidney failure	Qualitative- focus groups	Various methods of multidimensional assessments (e.g., functional, cognitive, psycho‐social, and somatic status)	Nephrologists, geriatricians, nurse practitioners, dialysis nurses, social workers and dieticians	Nephrologist and Geriatrician
Cravens et al. 2005. United States [38]	Home-based comprehensive assessment of rural elderly persons: the CARE project	Older community residents aged ≥ 75 years	Physicians and nurse practitioners (*n* = unknown)	In-home	To develop and pilot a model of rural home-based CGA to determine whether successful urban models can be adapted to rural areas	Mixed-methods. Qualitative Interviews. Immersion-crystallization approach to content analysis,	Multidisciplinary CGA led by a remote geriatrician. An in-home comprehensive assessment was completed by a trained nurse which was proactive and goal orientated. The social worker contacted patients remotely for additional information. The MDT held weekly meetings whereby a problem list and recommendations were formed	Geriatrician, nurse, administrator, and a social worker	Two geriatricians involved. The nurse was trained to complete the CGA components by a project geriatrician
Donaghy et al. 2023. United Kingdom [37]	General practitioner-led adapted comprehensive geriatric assessment for frail older people: a multi-methods evaluation of the ‘Living Well Assessment’ quality improvement project in Scotland	Older people living at home with moderate or severe frailty	General practitioners (*n* = 10)	In-home and then remotely due to COVID-19 restrictions	(1) To evaluate the impact of the LWA quality improvement project in primary care from the General practitioners’ and patients’ perspectives. (2) To determine whether there was a preference in the methods of delivery of the Living Well Assessment (CGA)(face to face and remote [telephone or video])	Mixed-methods-survey, interviews and focus groups	One-hour face-to-face assessment led by a General practitioner guided by a checklist. Referrals to other members of the MDT were made if necessary. An MDT meeting was held once a month for complex patients. Home assessments had to be changed to remote (video/telephone due to COVID-19 restrictions)	General practitioner	Participating General practitioners received training on carrying out the assessment from the project lead, who also received training from a practice in Scotland
Ericsson et al. 2021. Sweden [43]	“To be seen” – older adults and their relatives’ care experiences given by a geriatric mobile team (GerMoT)	Community-dwelling older people aged ≥ 75 years who have had 3 or more visits to the emergency care unit within the past 18 months and have ≥ 3 different diagnoses	Total sample *N* = 33. Older adults (*n* = 22) Relatives/caregivers (*n* = 11)	In-home and OPD in acute hospital	To obtain a better understanding, from the patients’ perspective, the experience of receiving CGA for both the participants and their relatives	Qualitative. Semi-structured qualitative interviews. Inductive qualitative content analysis	Individualised holistic interdisciplinary CGA including future care plan and follow-ups. An initial home visit is carried out by a nurse. A clinical pharmacist carried out a drug review. An out-patient medical assessment is carried out by a physician. Interdisciplinary meetings were held twice weekly where the patient's assessments were discussed and an individualised plan of care was made	Nurses, physicians, a physiotherapist, an occupational therapist, a pharmacist and a social worker	Not reported
Gardner et al. 2019. United Kingdom [34]	Comprehensive Geriatric Assessment in hospital and hospital-at-home settings: a mixed-methods study	Older people after an acute medical event, who were not severely unwell	Focus groups: Older people (*n* = 8) Caregivers (*n* = 3) Relatives (*n* = 3). Semi-structured interviews: HCPs (*n* = 11)	In-home	To define and describe the structure, content and delivery of the CGA as practised in hospital and hospital-at-home-based settings, from the perspective of health-care professionals who deliver it and patients and caregivers who experience this type of health care	Mixed-methods. Comparative case study- focus groups with older people, semi-structured interviews with HCP's. Framework approach to comparative analysis	A multidomain medical and therapeutic service provided to patients at home. Medical care and acute nursing was provided for up to 2 weeks, while rehabilitation therapy was available for up to 6 weeks	Hospital at Home 1: geriatricians, nurses with expertise in health care for older people, physiotherapists, occupational therapists, therapy assistants, pharmacists, a social worker and mental health liaison member. Hospital at Home 2: nurses, geriatricians, mental health specialist nurse and a pharmacist	Geriatrician involved
Ibrahim et al. 2022. United Kingdom [35]	The feasibility and acceptability of assessing and managing sarcopenia and frailty among older people with upper limb fracture	People aged ≥ 65 years with an upper limb fracture attending fracture clinics with sarcopenia and/or frailty	Total sample *N* = 22. Older people (*n* = 13) Orthopaedic consultants (*n* = 2) Nurses (*n* = 3) Geriatric practitioners (*n* = 4)	In-home	To evaluate the feasibility of assessing sarcopenia and frailty among people aged 65 + years attending fracture clinics with an upper limb fracture	Mixed-methods. Semi-structured interviews. Inductive thematic analysis	The majority of CGAs were conducted by geriatric. This involved a comprehensive assessment and MDT health and social care management pathway based upon individual assessment findings	Geriatrician and physiotherapist	Geriatrician involved. No other specific training involved
Junius-Walke et al. 2019. Germany [41]	How older patients prioritise their multiple health problems: a qualitative study	Older people in general practices aged ≥ 70 years	*N* = 34 older people	OPD in General Practice	To explore what underlying reasons patients have when they assess the importance of their health problems	Qualitative. Semi-structured interviews. Content analysis	Multicomponent assessment of health conditions and activities of daily living in the domains of function, social health, medical problems, mood, life-style, immunization, medication, cognition. Patients were presented with their findings after the CGA	Not reported	Not reported
King et al. 2017. New Zealand [42]	Implementation of a gerontology nurse specialist role in primary health care: Health professional and older adult perspectives	(1) Older adults aged ≥ 75 years who were enrolled in one of the 3 primary healthcare practices at risk of health and functional decline	Total (*n* = 11). General practitioner 's (*n* = 3) Nurse (*n* = 1) Hospital-based gerontological nurse specialist (*n* = 1) Primary healthcare gerontological nurse specialist (*n* = 1) Older people aged ≥ 75 years (*n* = 5)	In-home	To explore the new primary healthcare gerontological nurse specialist role from the perspectives of older people and health professionals	Qualitative. Semi-structured interviews. General descriptive inductive analysis	An in-home comprehensive holistic assessment targeting functional ability, cognitive impairment and depression with care co-ordination procedures was carried out by the Primary healthcare gerontological nurse specialist	Primary healthcare gerontological nurse specialist	The Primary healthcare gerontological nurse specialist received upskilling and mentorship as well as weekly case conferences and education sessions from the hospital-based specialist gerontology team
Mäkelä et al. 2020. United Kingdom [36]	The work of older people and their informal caregivers in managing an acute health event in a hospital at home or hospital inpatient setting	Older people aged ≥ 65 years who presented to the hospital acute assessment unit	Total sample *N* = 63. Older people (*n* = 15 who received hospital at home) Caregivers (*n* = 12 for patients who received hospital at home)	In-home	To explore the work of patients and caregivers at the time of an acute health event, the interface with health professionals in hospital and Hospital at Home and how their experiences related to the principles that underpin CGA	Qualitative. Semi-structured interviews. Normalisation process theory analysis	Geriatrician-led admission avoidance hospital at home with CGA. This involved provision of healthcare by MDT members including MDT meetings and daily virtual ward rounds and direct access to elements of acute hospital care	Geriatrician, doctors, nurses, physiotherapists and occupational therapists and referral to other services if required	Geriatrician involved
Rietkerk et al. 2019. Netherlands [30]	Explaining experiences of community-dwelling older adults with a pro-active comprehensive geriatric assessment program—a thorough evaluation by interviews	Home-dwelling frail older people aged ≥ 65 years	Older people (*n* = 25)	In-home or in OPD in General Practice	To explore and explain experiences of older adults who participated in a pro-active outpatient CGA program	Qualitative. Semi-structured interviews. Thematic analysis and cross-case analysis	The CGA included a multidomain assessment exploring psychological, social or functional needs. Additional allied health professional services were also offered when required. Individualised person-centred goals were devised from assessment findings. Written recommendations were offered to the older people and their general practitioners	Geriatric nurse or geriatric care physician. Other allied health professionals if required	Healthcare providers were trained in motivational interviewing. Geriatric nurse and geriatric care physician involved
Silverman et al. 1994. United States [39]	Geriatric Assessment: Inisde the black box	Older adults aged 65–90	Older people (*n* = 19) (*n* = 16 accompanied by a family member) HCPs (*n* = 22)	OPD in acute hospital	(1) To describe the treatment setting by identifying the similarities and differences in the four Geriatric Assessment Units (2) To describe and analyse the responses of providers, patients and family members to the CGA	Qualitative- process evaluation. Interviews. Analysis not clear	Not reported	Geriatrician, a geriatric social worker and a nurse	Geriatrician involved
Stijnen et al. 2014. Netherlands [31]	Nurse-led home visitation programme to improve health-related quality of life and reduce disability among potentially frail community-dwelling older people in general practice: a theory-based process evaluation	Potentially frail community-dwelling older people aged ≥ 75 years	Practice nurses (*n* = 13) General practitioners (*n* = 14) Older people (*n* = 17)	Home-based CGA	To examine (1) the extent to which the 'Getting OLD the healthy way' home visitation programme was implemented as planned in general practices, and (2) the extent to which general practices successfully redesigned their care delivery	Mixed methods. Semi-structured interviews. General inductive approach and conventional content analysis	A home-based CGA conducted by a practice nurse in collaboration with a General Practitioner and multidisciplinary intervention and follow-up was conducted. More elaborate assessments could be completed if deemed appropriate	Practice nurse and General practitioner	Not clearly reported. Practice nurses completed two-day training session that focused on gaining knowledge and skills to carry out the home visitation programme
Voorend et al. 2021. The Netherlands [32]	Perspectives and experiences of patients and healthcare professionals with geriatric assessment in chronic kidney disease: a qualitative study	Older adults ≥ 65 years living with end stage kidney disease	Six focus groups, *N* = 47. Older adults (*n* = 18) Caregivers (*n* = 4) Healthcare professionals (*n* = 25)	(1) out-patient clinic (2) home visit with telephone follow-up and (3) out-patient clinic	To explore perspectives and experiences of patients and professionals with geriatric assessment in the care for older (≥ 65 years) patients approaching end stage kidney disease, and to identify benefits, facilitators and barriers for implementation into routine nephrological care	Qualitative. Semi-structured focus groups. Inductive thematic analysis	(1) a yearly one-hour geriatric assessment in routine care for patients approaching end stage kidney disease performed in a university hospital conducted by a nurse practitioner or practice nurse (2) a three-hour geriatric assessment for patients approaching end stage kidney disease in a study setting conducted by a research nurse (3) a single-time point geriatric assessment among patients starting with or withholding from dialysis conducted by a nurse practitioner	Not reported	Not reported

#### Step 4

This stage involved determination of the key second-order interpretations and consideration of how the identified key concepts were related between studies. A grid format was used to categorise key concepts identified relevant to the research question in each study and juxtapose them against one another. This was completed by CH and reviewed by CF and KR.

#### Step 5

In this step, a reciprocal translation analysis was conducted. Although differences between the reports of various stakeholders were an important finding (for example differences between patient and HCP experiences), within stakeholder groups accounts were directly comparable, thus both refutational and reciprocal translation was possible. This phase was conducted by CH and reviewed by CF and KR. Constant comparisons were made highlighting similarities or differences between second-order constructs, from which over-arching concepts were developed. Important second-order concepts for each stakeholder group were compared with concepts within that stakeholder group across all included papers in turn, thus were translated into one another. Team discussions were held ensuring collaborative interpretation of concepts. CH maintained a reflective journal in order to ascertain the researchers ‘place in the text’ from a theoretical point of view during the analysis phase [18, 28]. Translation of concepts from one study into another allowed the team to generate third-order concepts which represented more than one study [18].

#### Step 6

In this phase, we were not able to generate a line-of-argument from third-order constructs as both refutational and reciprocal translation had occurred.

#### Step 7

The final step involved writing up the synthesis results for dissemination using the eMERGE checklist to enhance transparency in reporting [17] (Additional file 1).

#### Patient and Public Involvement (PPI)

The Ageing Research Centre at the University of Limerick established a Public and Patient Involvement (PPI) stakeholder panel of older adults and family caregivers in 2020 [29]. This group meet with the research team every 4–6 weeks and have consulted on this study from inception. The PPI panel were also involved in interpretation of study findings. When the overarching themes (third-order constructs) were developed, a 2-h meeting was arranged with seven older adults / family caregiver members of the PPI panel. The meeting was facilitated by four members of the research team. With regards to this study, the group agreed that the third order constructs reflected their own, and family members experiences, namely, that a single point of contact for older adults receiving care from multiple healthcare professionals was invaluable. There was a consensus that there needs to be an enhanced effort by HCPs to deliver appropriate information to older adults, as they may have difficulties such as hearing impairments, visual impairments or fatigue, especially when medical attention was warranted. Further discussion with one PPI member regarding the value of home-based health services identified how this approach enabled a more realistic social and environmental assessment of the person’s needs, while overcoming logistical issues around transport to healthcare appointments for people who were unable to access same.

## Results

### Search outcomes

**Fig. 1:**
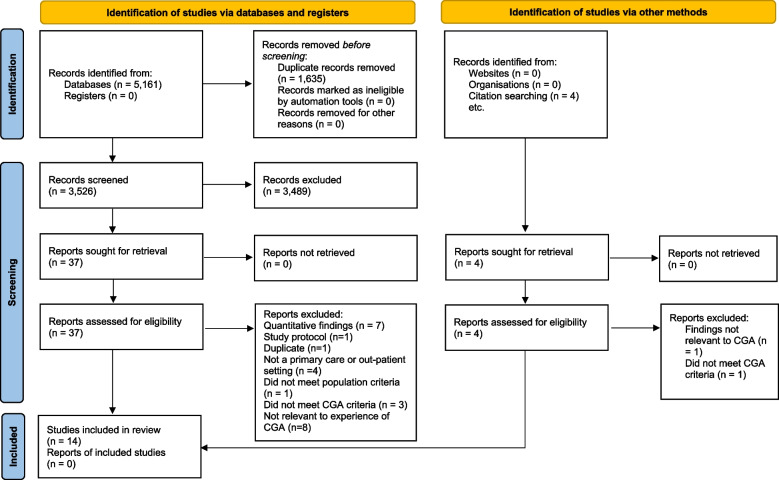
Flow of search, identification and selection process

The systematic search identified 5,165 studies with 1,639 duplicates removed. Titles and abstracts were screened and a further 3,487 studies were removed based on eligibility criteria. Full-text screening was completed on 41 studies leading to the inclusion of 14 studies in the final review (Fig. 1).

### Characteristics of included studies

Details of the 14 included studies are outlined in Table 1. Four studies were conducted in both the Netherlands [30–33] and the United Kingdom [34–37]. Two studies were conducted in the United States [38, 39], two were conducted in Germany [40, 41], one was conducted in New Zealand [42], and one in Sweden [43]. Six studies employed a mixed-methods approach [31, 34, 35, 37, 38, 40] while eight of the included studies employed exclusively qualitative methods [30, 32, 33, 36, 39, 41–43]. The CGA was completed in the older person’s home in six studies [31, 34–36, 38, 42], an out-patient setting in general practice in three studies [30, 40, 41], an out-patient setting in acute hospitals in three studies [32, 33, 39], the older persons home which then converted to virtual assessments due to the impact of COVID-19 in one study [37] and within the home and OPD in acute hospital setting in one study [43].

With regards to population, three studies were conducted with HCPs only [37, 38, 40], five with HCPs and older adults [31, 33, 35, 39, 42], two with HCPs, older adults and caregivers [32, 34], one with older adults and caregivers [36] and three with older adults only [30, 41, 43]. One study did not report the number of participants involved [38]. The other 13 studies reported on the experiences of 155 HCPs, 194 older adults, 23 caregivers and 3 close relatives. Four studies identified older adults living with frailty for inclusion in the study [30, 31, 35, 37], five studies identified older adults at risk of experiencing functional or health decline [34, 36, 39, 42, 43], while three studies included all community-dwelling older adults [38, 40, 41]. Two studies specifically explored participants experience of CGA for older adults living with kidney disease [32, 33].

While the team composition were heterogenous across studies, five studies outlined the involvement of a geriatrician within the process of care [34–36, 38, 39], one study outlined the involvement of a consultant physician [43], three studies clearly reported further specialist training in geriatric care among a member of the team [30, 33, 42] and nine studies involved health and social care professionals within the team (seven of these including allied health professionals) [30, 31, 33–36, 39, 42, 43].

### Quality appraisal

Findings from the quality appraisal are outlined in Additional file 3. All included studies reported a clear statement of the aim and appropriate methodology. Two studies reported insufficient detail regarding the recruitment process [38, 40]. Notably, only five studies adequately addressed the relationship between researcher and participant [30–32, 42, 43], and two studies provided insufficient detail of how data analysis was carried out [38, 39].

### Synthesis

The analysis produced four themes (third-order constructs): (a) CGA is experienced as a holistic process, (b) The home environment enhances CGA, (c) Sufficient time, a proactive approach and interprofessional communication enable CGA in the community and, (d) Divergent experiences of the meaningful involvement of older adults, caregivers and family in the CGA process. An overview of the contribution from studies to each theme is outlined in Table 2.

**Table 2:** Contribution of included studies towards themes

Citation	CGA is experienced as a holistic and personalised process	The home environment enhances CGA	Sufficient time, a proactive approach and interprofessional communication enable CGA in the community	Divergent experiences of the meaningful involvement of older adults, caregivers and family in the CGA process
Cravens et al. 2005. [38]	X	X	X	
Barkhausen et al. 2015. [40]			X	
King et al. 2017.[42]	X	X	X	X
Rietkerk et al. 2019. [30]	X		X	X
Stijnen et al. 2014. [31]	X	X	X	X
Junius-Walke et al. 2019. [41]				X
Ericsson et al. 2021. [43]	X	X	X	X
Ibrahim et al. 2022. [35]	X		X	
Mäkelä et al. 2020. [36]	X	X	X	X
Gardner et al. 2019. [34]	X	X	X	X
Voorend et al. 2021. [32]	X	X	X	X
Silverman et al. 1994. [39]	X	X	X	X
Donaghy et al. 2023. [37]	X	X	X	X
Berkhout‐Byrne et al. 2023. [33]	X			X

### CGA is experienced as a holistic process

A holistic approach to CGA was reported in multiple studies as having a positive impact on older adults’ experiences of CGA and satisfaction [30–35, 37–39, 42, 43]. Both older adults and HCPs recognised the importance of focusing on physical health alongside social, quality of life and other domains [30, 32, 33, 37]. Older adults spoke about how a broader holistic approach enabled them to feel seen as a whole individual [30, 32]:

*“Because it has to do with being seen. That you really see the other person as a whole individual. That you are not just that pelvis, or that arm that is broken, or whatever, but that you see the human being. That is the most important thing for me…That you are not just an ailment that needs to be resolved. But that you are seen as a human being”* [30].

This holistic approach to CGA was credited by HCPs as capturing a broader overview of the older person’s presentation:

*“In terms of looking at caring for someone at home you have to have that comprehensive overview. You can’t possibly manage someone without knowing as much about them as possible. That obviously isn’t just medical, it is social aspects as well”* [34]*.*

A comprehensive approach was also reflected in accounts of the pre-emptive role of CGA, where older adults felt the CGA offered an opportunity to discuss health problems or concerns they had not yet sought or found help for, or to discuss concerns that their General Practitioner (GP) or other HCPs would not usually have time to discuss [30, 31, 37, 42]. An example of CGA enabling discussion of a problem for which an older adult had not yet found help was reported in a study of the outpatient pro-active assessment program Sage-atAge;

*“I went to [the ophthalmologist] and then they said “We can’t do anything for you anymore”. After two operations, on both eyes. {} {Then the Sage-atAge nurse advised to go to a vision-aid centre}. {} Then I thought, well, isn’t this something. You go to [the hospital], and they did not know what to do with me.”*[30].

The comprehensive nature of CGA was also described in some studies as ‘proactive’. Patients and staff valued proactive care as it “*made a positive impact to the older person’s physical environment and physical health*” [42], while also had the positive effect of delivering unanticipated help to older adults [30]. The shift in delivery of care from a reactive *‘once off’* healthcare intervention towards a more proactive approach in the community was described as difficult for many HCP’s, particularly GPs in one study [31]:

*“GPs were not used to approaching older people in a proactive way. They usually offer care and/or treatment upon request, whereas PNs are more familiar with delivering preventive care”* [31].

The delivery of care by highly skilled HCPs was reported as a key factor to the successful implementation / delivery of CGA [30–34, 39, 42]. Older adults acknowledged and appreciated when HCPs were attentive, supportive and reassuring [30] and provided clear education and demonstrated advanced critical thinking that allowed for a holistic assessment and anticipation of what the older adult needed ahead of time [30, 37, 42].

### The home environment enhances CGA

The value of conducting CGA in the home environment was discussed in ten studies [31, 32, 34, 36–39, 42, 43]. HCPs in six studies reported that in-home CGAs are preferable to in-clinic assessments to allow for a more in-depth assessment, including an environmental and social observation of older adults, that couldn’t otherwise be assessed in a healthcare setting [31, 32, 34, 37, 38, 42]:

“*You see a lot during a home visit, for example: you observe the interaction between husband and wife, and between parents and concerned children” *[32].

The home-based CGA was described as facilitating integration of care at the system level, where those who completed the in-home assessments provided detailed feedback to other care providers and stakeholders [42]. Both HCPs and older adults described home-based assessments and having sufficient time to enable development of rapport and disclosure of information by the older adult:

*“... she has the time to spend with them in their own homes, so they will chat more to her” *[42].

HCPs also described how the home assessment led to older adults being more empowered, allowing them to have a more active role in the self-management of their health conditions [31, 38, 42]:

*“I get the opportunity to enhance and develop their self-management skills which for chronic care conditions is a significant skill that needs to be optimised so that they can manage as well as they can” *[42].

HCPs described CGA in the home as personalised to the older person’s needs, aiding the establishment of functional goals, facilitating family engagement in the CGA and improving access to healthcare [34, 37].

Older adults described positive feelings associated with a home-based assessment such as feelings of relaxation, support and security [31, 34, 36, 39, 43] and increased accessibility of healthcare [31, 39, 43]. These experiences at home were contrasted with CGA in the acute out-patient settings where functional difficulties often portrayed by older adults proved a barrier to successful CGA, especially where the CGA required multiple visits [39]. CGA at home was also seen as superior to virtual assessment via telephone [43]:

*“Something that was appreciated was that the examinations could be done at home if the person had difficulty travelling to the clinic. Some persons put forward that it was better to get a home visit because it was sometimes difficult to gather one’s thoughts during a phone call” *[43].

Older adults reported a desire to avoid a hospital stay and expressed preferences to remain at home [36]. This was further expressed by caregivers where they reported valuing home-based care in order to avoid additional distress associated with unfamiliar hospital-based surroundings, especially when the older adult was experiencing confusion or symptoms of delirium [36].

“ *Aisla’s daughter valued avoiding additional distress from the unfamiliar surroundings of hospital, describing her own strategies for managing when her mother was being treated for delirium at home: “There’s bits where this isn’t her house and then all of a sudden, yeah, it is. . . if you’re here and you get confused that this isn’t the house, then we can talk about familiar things and it’s almost like you’re back in the room again*” [36].

### CGA in the community is enabled by interdisciplinary collaboration

Interdisciplinary collaboration within teams and across care settings, were highlighted by stakeholders as a facilitator of CGA. HCP’s and older adults described interdisciplinary communication across disciplines and settings as enabling CGA delivery and success [30–34, 36, 39, 42].

*“Any member of the team may carry out the initial CGA assessment, although specific disciplinary expertise might be drawn upon depending on patient need. A nurse explained it in the following terms: “It is a bit like a jigsaw . . . So we can actually work quite independently as practitioners, the therapy team, Age UK, so we all go off and do our own priorities in our own direction but then bring it back to the team” *[34].

In one study where home based CGA by a practice nurse followed by targeted interdisciplinary care was initiated for older people, practice nurses described “extending their network of professionals or disciplines involved in care for older people and they used their network to a greater extent” in this new model of care [31].

HCP communication and engagement across the acute and primary care setting was described in the study by King and colleagues’ as enhancing delivery of CGA [42]. Interdisciplinary collaboration was frequently described as improving integration of care for older adults [30, 34, 36, 42]. Interdisciplinary collaboration was seen as a way to reduce the “*risk that the fragmentation of knowledge will not be brought together into a decision at the multidisciplinary team meeting*” [32]. One study noted that the current lack of structured MDT meetings in the community setting was a potential barrier to optimal MDT discussions [31].

Older adults and HCP’s discussed care-coordination as an inherent part of CGA and care-coordination was valued by older adults and seen to enhance delivery of integrated holistic care [30, 32, 36, 42, 43]. One paper outlined that expert care co-ordination was seen as an enabler to integration and timeliness of healthcare services and reduce fragmentation of services for older adults across different organisations and settings while also empowering older adults to self-manage chronic diseases [42]:

*“She knew her subjects and knew what she could recommend as good for you. She put me on to several [other services] that were able to help me. I was thoroughly satisfied, I’d be quite happy if she came back” *[42].

### Divergent experiences of meaningful involvement of older adults, caregivers and family in the CGA process

Clear communication with older adults was highlighted by both HCPs [32, 34, 42] and older adults [30, 33, 34, 36, 39, 42] as supporting a positive experience of CGA. HCPs viewed communication as key to the facilitation of CGA [34], while older adults spoke more in terms of how clear communication assisted in helping to feel prepared for the CGA [32] and led to better satisfaction with care [30].

High quality patient engagement in CGA occurred where older adults were aware of the purpose of CGA [39, 42, 43] and plan arising from CGA and had opportunities for discussions with HCPs [43]*.*

HCP’s also noted the impact of communication with older adults about the purpose of CGA on older adult satisfaction with CGA [30, 32]:

*“A clear explanation of the purpose and outcomes is important for patients” *[32].

Sufficient time was reported by HCP’s and older adults as essential to enable communication and collaboration. Older adults valued the additional quality time allotted to them by HCPs as part of the CGA leading to enhanced, patient experience, stronger rapport building and trust which supported patient disclosure [31–34, 42]:

*“Patients appreciated the attention during geriatric assessment for multiple aspects of health and daily functioning. They particularly valued the (extra) time and attention they received from professionals. Consequently, patients were able to share more fears and concerns about treatment choices*” [32]*.*

Although providing more time to complete a CGA compared to usual care is one of the more ubiquitous factors required to ensure successful delivery of CGA, the time investment required [31, 37, 40] in addition to a lack of budget [32, 37] and staffing [31, 37, 40] were noted as potential barriers to carrying out the home-based CGA visits by some HCPs:

*“Barriers for continuing the home visitation programme over time were the lack of an adequate reimbursement by health insurers of the costs of care for older people and the overall time investment of the home visitation programme” *[31].

While it was clear all three stakeholder groups appreciated the importance of communication in the CGA experience, it is also clear that there was a need for enhanced communication efforts [30, 32, 34, 36, 39].

Caregivers found that a lack of sufficient communication from HCPs often resulted in disrupted rapport building, leaving the patient feeling not fully informed about CGA:

*“Lack of continuity had disrupted rapport-building when different team members had come to the home and could be compounded by an approach of ‘being informed’, rather than ‘being included’, within discussions” *[34].

In one study insufficient feedback to older adults on test results were highlighted:

*“Patients mentioned that they did value discussing personal results and implications, but that in some hospitals feedback on results was lacking. Shortcomings in communication about the purpose in routine care were acknowledged by some professionals ” *[32]*.*

The lack of opportunity for patients, caregivers, and family to be involved in the CGA was discussed by older adults and caregivers in two studies [34, 36]. While HCPs spoke of the importance of involving family and caregivers in the CGA [32, 34, 37, 39, 42], highlighting the value this makes to the CGA and care plan for the patients, family members and caregivers felt that they were not presented with the opportunity for engagement and involvement, impacting the decision-making process for patient care planning [34, 36]:

*“Patients and caregivers did not recognise CGA as a process of assessment and planning that involved them. Family care-givers, even when involved in providing personal care and having daily contact with their relative, perceived they had not been invited to contribute to assessments on acute units, and that their knowledge of cognitive, communicative and physical functioning could have informed decision-making” *[36].

In some instances there appeared to be an assumption from some HCPs that caregivers were involved in the assessment, yet little consideration appears to have been given to actively facilitate and engage with this group [34]*.* Limited opportunity for caregivers to engage in the assessment described in the above quote stemmed from lack of effective communication and rapport between HCPs and caregivers. One study outlined how a family member who wanted to provide information as part of the assessment did not do so as they felt they were being a “nuisance” to the HCPs:

*“Caregivers striving to support their relative at home had not always felt able to raise the topic or ask about additional assistance if an opportunity for discussion had not been created by professionals within interactions” *[34].

HCPs in another study reported that the presence of the primary caregiver during the CGA helped the assessor gain more information about the older person, while also allowing for an informal assessment of caregiver stress, which could ultimately lead to premature placement of older adults in residential settings [42]. Caregiver / family involvement was also reported to potentially support the treatment plan arising from CGA:

*“For the clinicians, the families’ involvement can have distinct advantages. Families may be supportive of the treatment plan and help to facilitate it.” *[39]

Discharge planning was a particular element of the CGA pathway that was identified by caregivers as a missed opportunity for their engagement [34, 36]:

*“A key concern raised by caregivers… was insufficient involvement in determining discharge arrangements. … conflicting communication from the team about discharge plans and lack of family involvement had raised anxiety” *[34].

## Discussion

To the best of our knowledge, this is the first meta-ethnographic study to systematically synthesise the evidence of older adults’, caregivers’ and HCPs’ experiences of CGA in community and OPD settings. The findings suggest that CGA, for the most part, is experienced positively by older adults, caregivers and HCPs. CGA in the home environment was valued for various reasons; it allowed for a more in-depth assessment according to HCPs and it led to empowerment of older adults, enhanced feelings of security and support while increasing accessibility to healthcare. Findings revealed the holistic approach to care afforded older adults the opportunity to discuss health problems that they would ordinarily not have time to discuss during other healthcare encounters. Important facilitators of this holistic assessment were sufficient HCP time, care and service integration and interprofessional communication. Ideally, CGA as a process involves older adults and caregivers as partners. While it is evident that all stakeholders appreciate the importance of communication and older adult / caregiver involvement in CGA, it is also clear that enhanced communication efforts are required to realise this ambition fully.

We found that across stakeholder groups there was consensus that CGA enabled a holistic assessment of older adults needs in community and OPD settings. This is an important finding as many older adults have complex and heterogeneous needs [44], with 17.4% of the community-dwelling population older adult population living with frailty [45]. Furthermore, the WHO promote a comprehensive assessment alongside care co-ordination as integral to the successful delivery of integrated clinical care to older adults, particularly within the community setting [7]. A recent Cochrane review of CGA for community-dwelling older adults in the community setting found reductions in unplanned hospital admission but little change in QoL and function among the cohort, although it was acknowledged that there was heterogeneity and inconsistencies across studies [9]. Of the 21 studies included in the Cochrane review, the majority (*n* = 12) were delivered in an OPD setting alongside varied models of CGA, but none of the included studies compared OPD only interventions to domiciliary care. Our synthesis demonstrates that stakeholders experienced home as a preferred environment for CGA as it enabled both enhanced feelings of security and comfort for older adults while enabling a more comprehensive assessment by HCP’s. These findings align with a qualitative evidence synthesis exploring older adults’, caregivers’ and HCPs’ experiences of CGA in the acute setting whereby the hospital environment was identified as a suboptimal setting for addressing inclusive goal setting and care planning (O’Shaughnessy, personal communication). Future trials of CGA outside the hospital setting should address the limitations highlighted by Briggs and colleagues in their Cochrane review [9], particularly relating to the impact of domiciliary versus OPD CGA and the impact of community CGA on older adult satisfaction and quality of life [9]. Given the varied models of CGA operationalised in the studies included in the Cochrane Review [9], further research is needed to establish which, if any model is most effective and the advantages / disadvantages of various models. Detailed reporting of the CGA model in future trials in community or OPD settings would be of value. Future consensus based research methods may also be of value to gain consensus on the operational model and outcome measurement of CGA in community and OPD settings. Future research should also explore the implementation of standardised assessment protocols for CGA, as these types of protocols have been shown to address regional practice variation and improve patient outcomes [46].

CGA requires a multidisciplinary team review that includes doctors, allied health professionals, nurses and pharmacists focussed on a holistic assessment to inform an individualised, pro-active and coordinated care plan [47], from which a ‘roadmap for unified action’ is developed [7]. In the studies included in this review CGA was conducted by varied combinations of personnel. Only four of the 14 included studies included a medical, allied health and nursing assessment, seven involved allied health professionals, one study involved nursing only, one included nursing and the general practitioner only and three studies did not report the team composition. Increased healthcare staff recruitment is required to support government policy to shift care to the primary care and community setting in countries such as Ireland with limited workforce being recognised as a barrier to the delivery of integrated care [48]. Suggestions for how to address the primary care workforce shortage in community based geriatric healthcare settings include elevating the role of nurses and caregivers, shifting towards more integrated and collaborative approaches to care where medical, nonmedical, social service and community providers all play an active role [49].

The central role of good care coordination and collaboration among stakeholder groups and across healthcare professionals in the delivery of CGA was reflected strongly in the current findings. The WHO defined care coordination as ‘a proactive approach to bringing together care professionals and providers to meet the needs of service users to ensure that they receive integrated, person-focused care across various settings’ [50]. We found that older adults wanted care coordinator input because it facilitated timely access to care and improved transitions of care between services which are concerns raised by older adults living with chronic diseases [51]. This is difficult to achieve within fragmented care systems where there is resistance to a proactive approach to care by older adults and healthcare professionals [15]. Despite this, findings from this synthesis revealed that CGA inclusive of holistic assessment, tailored interventions, clear communication with all stakeholders and coordination of healthcare services has the potential to be experienced positively by older adults and their caregivers.

Older adults and caregivers should be partners in care and decision-making with HCPs to enable person-centred care [52, 53]. A central theme among the included studies in this synthesis was an idealisation of older adults, caregivers and HCPs working as partners during the CGA process. Our findings suggest that clear communication facilitated older adults to have a more active role in the self-management of their conditions. However, we also found that missed opportunities for older adults and caregivers involvement in CGA in some studies. Previous research has shown that although both families and HCPs value good communication, what they perceive good communication to be may differ [54]. Our findings also lend support to the idea that HCPs may make assumptions about what person-centred care or shared decision-making means. Research has found that shared decision making in older adults living with frailty when making healthcare choices is an iterative process that involves individualised and person-centred communication between HCPs and patients that begins at the start of the consultation and ends with a reflection on the process [55]. It also includes counselling patients on important health issues, advance care planning, the importance of outlining the patient’s decision-making capacity and their values and care goals [55].

Our findings reflect those of Garrard and colleagues’ where miscommunication and lack of patient education led to patients being less compliant with their healthcare management recommendations [56]. Improved communication was highlighted as a requirement to support older adults to become more actively involved in their care, with practitioners describing the benefits of patient involvement as increasing patient engagement and understanding in their care pathway, as outlined in the European Commission report on patient involvement [54]. Furthermore, a previous qualitative analysis of older adults living with frailty experiences of CGA enabled them to feel “*respected as a person”* when invited to participate in the decision-making process [57]. Our findings support these considerations as both older adults and caregivers report the need for more opportunities for engagement within the CGA process.

Collaboration between care providers is essential and should be a key priority for the successful delivery of care to older adults [7, 58]. Despite this, our findings suggest that fragmented care services act as a barrier to clear communication pathways between care providers. These insights are further supported by qualitative findings by Sum and colleagues whereby a lack of communication between HCPs who carried out geriatric assessments resulted in a potential for reduced patient engagement and adherence to care plans by patients [15]. The main barrier to effective communication as described by both older adults and HCPs is the time available to HCPs as outlined in the European commission report for patient involvement [54]. Our findings mirror this as while having sufficient time was described as an enabling factor for completion of a comprehensive assessment, the time investment, budget and staffing shortages were perceived as potential barriers to CGA. Future trials of CGA outside hospital settings would benefit from integrating the findings of this synthesis into the refinement of the intervention in line with the Medical Research Council guidance on the design and evaluation of complex interventions [59]. This would ensure that future trials of CGA address the barriers to meaningful involvement of older adults and caregivers in CGA.

### Strengths and limitations

To the best of our knowledge, the current meta-ethnography is the first to systematically identify and synthesise various stakeholder experiences of CGA in community and out-patient settings. Meta-ethnography is an interpretive approach to analysis, and other researchers may have drawn different theories from the data. However, the broad search string applied enhanced the rigor of study identification. Two authors (CH and CF) engaged with the included studies over a long period of time, engaged in reflective writing to support researcher reflexivity, adhered to reporting guidelines in the conduct and reporting of the review and noted the number of studies contributing to each third-order construct enhancing rigor and transparency of the findings. Consideration of researcher quotations and contrasting stakeholder experiences were also included within each construct identified. The research team comprises HCPs and researchers working in clinical and academic settings. CH is a PhD candidate and physiotherapist based in the community setting who has completed specialist training on meta-ethnography. Multiple members of the research team have extensive qualitative research experience and almost all members of the team have experience of conducting CGA as a HCP in the community or in acute settings. Because we were considering accounts of HCPs, caregivers, and older adults, we reflexively considered how our professional backgrounds and experiences as HCP's may have influenced interpretation of findings through in-depth team discussions throughout the analysis process. Furthermore, all interpretations were checked against the original data and preliminary third order constructs were discussed with a PPI panel of older adults and family carers to further interrogate our interpretations.

However, limitations of this study may affect the generalisability of the findings. Firstly, samples are drawn from a mixture of community-dwelling older adults who are living with frailty, who are identified as required increased healthcare use, have a recent fracture, who are living with kidney disease and who are not identified as at-risk. The search strategy for this review was limited to the English language. Therefore, additional data may have been available from other studies not published in the English language. The methodological limitations of the included studies limit the synthesis findings. No study was excluded based on quality appraisal however only six of the fourteen included studies received a positive score for every criterion. Further exploration of caregiver experience is warranted as only three of the included studies had caregiver involvement. The duration of the CGA was not clear in included studies, which may enhance our understanding of stakeholder experiences depending on the level of care received.

## Conclusion

This synthesis found that CGA in community and OPD settings was positively experienced as a holistic process that was enabled and enriched by the context of the home environment and by communication and collaboration among stakeholders. However, we also found divergent perspectives on the meaningful involvement of older adults, caregivers and family in the CGA process. Healthcare professionals in the community should ensure meaningful involvement of older adults and their families or caregivers in the CGA process to ensure that their contributions are valued, and their concerns are addressed. Further robust trials of different models of community based CGA informed by the findings of this synthesis are warranted.

### Supplementary Information

**Additional file 1. **The eMERGe Meta‐ethnography Reporting Guidance.

**Additional file 2. **Search strategy.

**Additional file 3. **Results of CASP quality appraisal.

## Data Availability

The data analysed in the current study are available from the corresponding author on reasonable request.

## Abbreviations

WHO: World Health Organsiation
CGA: Comprehensive Geriatric Assessment
RR: Risk Ratio
CI: Confidence Interval
NH: Nursing Home
ED: Emergency Department
OPD: Out-Patient Department
QES: Qualitative Evidence Synthesis
HCP: Healthcare Professional
CASP: Critical Appraisal Skills Program
PPI: Public and Patient Involvement
GP: General Practitioner
