# Supplementary material for: Exploring stakeholders’ experiences of comprehensive geriatric assessment in the community and out-patient settings: a qualitative evidence synthesis
Source: BMC Prim Care. 2023 Dec 13;24:274. doi: 10.1186/s12875-023-02222-2 (PMC10717956; doi:10.1186/s12875-023-02222-2)
Supplement: Supplementary file 1 — Additional file 1. The eMERGe Meta‐ethnography Reporting Guidance. [file 12875_2023_2222_MOESM1_ESM.docx]

**Additional file 1-** The eMERGe Meta‐ethnography Reporting Guidance

| **No.** | **Criteria Headings** | | **Reporting Criteria** | | **Location in text** |
| --- | --- | --- | --- | --- | --- |
| Phase 1—Selecting meta-ethnography and getting started | | | | | |
| *Introduction* | | | | | |
| 1 | Rationale and context for the meta-ethnography | | Describe the gap in research or knowledge to be filled by the meta-ethnography, and the wider context of the meta-ethnography | | Page 4, 5 |
| 2 | Aim(s) of the meta-ethnography | | Describe the meta-ethnography aim(s) | | Page 5, 6 |
| 3 | Focus of the meta-ethnography | | Describe the meta-ethnography review question(s) (or objectives) | | Page 5,6 |
| 4 | Rationale for using meta-ethnography | | Explain why meta-ethnography was considered the most appropriate qualitative synthesis methodology | | Page 5 |
| Phase 2—Deciding what is relevant | | | | | |
| *Methods* | | | | | |
| 5 | Search strategy | | Describe the rationale for the literature search strategy | | Page 7 |
| 6 | Search processes | | Describe how the literature searching was carried out and by whom | | Page 7 |
| 7 | Selecting primary studies | | Describe the process of study screening and selection, and who was involved | | Page 7,8 |
| *Findings* | | | | | |
| 8 | Outcome of study selection | | Describe the results of study searches and screening | | Page 8, 18, 19, 20 |
| Phase 3—Reading included studies | | | | | |
| *Methods* | | | | | |
| 9 | Reading and data extraction approach | | Describe the reading and data extraction method and processes | | Page 9, |
| *Findings* | | | | | |
| 10 | Presenting characteristics of included studies | | Describe characteristics of the included studies | | Page 10-16,19 |
| Phase 4—Determining how studies are related | | | | | |
| *Methods* | | | | | |
| 11 | | Process for determining how studies are related | | Describe the methods and processes for determining how the included studies are related: - Which aspects of studies were  compared AND  - How the studies were compared | Page 17 |
| *Findings* | | | | | |
| 12 | | Outcome of relating studies | | Describe how studies relate to each other | Page 17, 20, 21 |
| Phase 5—Translating studies into one another | | | | | |
| *Methods* | | | | | |
| 13 | | Process of translating studies | | Describe the methods of translation:  -Describe steps taken to preserve the context and meaning of the relationships between concepts within and across studies -Describe how the reciprocal and refutational translations were conducted -Describe how potential alternative interpretations or explanations were considered in the translations | Page 17 |
| *Findings* | | | | | |
| *14* | | Outcome of translation | | Describe the interpretive findings of the translation. | Page 17 |
| Phase 6—Synthesizing translations | | | | | |
| *Methods* | | | | | |
| 15 | | Synthesis process | | Describe the methods used to develop overarching concepts (“synthesised translations”)  Describe how potential alternative interpretations or explanations were considered in the synthesis | Pages 17 |
| *Findings* | | | | | |
| 16 | | Outcome of synthesis process | | Describe the new theory, conceptual framework, model, configuration, or interpretation of data developed from the synthesis | Page 17, 20-29 |
| Phase 7—Expressing the synthesis | | | | | |
| *Discussion* | | | | | |
| 17 | | Summary of findings | | Summarize the main interpretive findings of the translation and synthesis and compare them to existing literature | Pages 29-33 |
| 18 | | Strengths, limitations, and reflexivity | | Reflect on and describe the strengths and limitations of the synthesis: - Methodological aspects—for example, describe how the synthesis findings were influenced by the nature of the included studies and how the meta-ethnography was conducted. -Reflexivity—for example, the impact of the research team on the synthesis findings | Page 33 |
| 19 | | Recommendations and conclusions | | Describe the implications of the synthesis | Page 34 |
