# Supplementary material for: Exploring stakeholders’ experiences of comprehensive geriatric assessment in the community and out-patient settings: a qualitative evidence synthesis
Source: BMC Prim Care. 2023 Dec 13;24:274. doi: 10.1186/s12875-023-02222-2 (PMC10717956; doi:10.1186/s12875-023-02222-2)
Supplement: Supplementary file 2 — Additional file 2. Search strategy. [file 12875_2023_2222_MOESM2_ESM.docx]

**Additional File 2. Search strategy**

| S1 | CGA OR Geriatric Assessment OR comprehensive geriatric assessment OR geriatric evaluation OR Geriatric consultation OR multidisciplinary assess* OR multi‐disciplinary assess* |
| --- | --- |
| S2 | Qualitative OR Experience* OR Perspective* OR case stud* OR interview* OR focus group* OR participant observation OR Naturalistic observation OR ethnograph* OR phenomenol* OR lived experience OR grounded-theor* OR thematic analysis OR Narrative analysis OR Discourse analy* OR field-notes |
| S3 | S1 AND S2 |
