# Supplementary material for: Exploring stakeholders’ experiences of comprehensive geriatric assessment in the community and out-patient settings: a qualitative evidence synthesis
Source: BMC Prim Care. 2023 Dec 13;24:274. doi: 10.1186/s12875-023-02222-2 (PMC10717956; doi:10.1186/s12875-023-02222-2)
Supplement: Supplementary file 3 — Additional file 3. Results of CASP quality appraisal. [file 12875_2023_2222_MOESM3_ESM.docx]

**Additional file 3. Results of CASP quality appraisal**

|  | **CASP Criterion 1** | **CASP Criterion 2** | **CASP Criterion 3** | **CASP Criterion 4** | **CASP Criterion 5** | **CASP Criterion 6** | **CASP Criterion 7** | **CASP Criterion 8** | **CASP Criterion 9** |
| --- | --- | --- | --- | --- | --- | --- | --- | --- | --- |
| **Citation** | **Clear statement of aim** | **Qualitative methodology appropriate** | **Appropriate research design** | **Sampling** | **Data collection** | **Research reflexivity** | **Ethical consideration** | **Appropriate data analysis** | **Clear statement of findings** |
| **Barkhausen et al. 2015** | Yes | Yes | Yes | Can’t tell | Yes | No | Yes | Yes | Yes |
| **Berkhout‐Byrne et al. 2023** | Yes | Yes | Yes | Yes | Yes | Yes | Yes | Yes | Yes |
| **Cravens et al. 2005** | Yes | Yes | Yes | Can’t tell | Can’t tell | No | Can’t tell | Can’t tell | Yes |
| **Donaghy et al. 2023** | Yes | Yes | Yes | Yes | Yes | Yes | Can’t tell | Yes | Yes |
| **Ericsson et al. 2021** | Yes | Yes | Yes | Yes | Yes | Yes | Yes | Yes | Yes |
| **Gardner et al. 2019** | Yes | Yes | Yes | Yes | Yes | Can’t tell | Yes | Yes | Yes |
| **Ibrahim et al. 2022** | Yes | Yes | Yes | Yes | Yes | Can’t tell | Yes | Yes | Yes |
| **Junius-Walke et al. 2019** | Yes | Yes | Yes | Yes | Yes | Can’t tell | Yes | Yes | Yes |
| **King et al. 2017** | Yes | Yes | Yes | Yes | Yes | Yes | Yes | Yes | Yes |
| **Mäkelä et al. 2020** | Yes | Yes | Yes | Yes | Yes | Can’t tell | Yes | Yes | Yes |
| **Rietkerk et al. 2019** | Yes | Yes | Yes | Yes | Yes | Yes | Yes | Yes | Yes |
| **Silverman et al. 1994** | Can’t tell | Yes | Yes | Can’t tell | Can’t tell | No | Yes | Can’t tell | Yes |
| **Stijnen et al. 2014** | Yes | Yes | Yes | Yes | Yes | Yes | Yes | Yes | Yes |
| **Voorend et al. 2021** | Yes | Yes | Yes | Yes | Yes | Yes | Yes | Yes | Yes |
